# Errata

**Published:** 1785

**Authors:** 


					p. 101, 1. i6,
for generatoni, read gcncratiom.

				

